# Ligature of External Iliac

**Published:** 1872-03

**Authors:** S. A. Freeman

**Affiliations:** A. A. Surgeon U. S. A.; Camp Wright, Cal.


ART. III.—*Ligature of External Iliac.* By S. A. FREEMAN, A. A. Surgeon U. S. A.

Private William Hedman, German, of Co. A. 12th U. S. Infantry, was admitted to Post Hospital, at Camp Wright, Mendocino County, California, on the morning of November 21st, 1871, suffering from what I at once diagnosed to be Aneurism of the Femoral Artery, at the terminus of the external Iliac. Had been on fatigue duty three days before, and while assisting in lifting curb from well undergoing process of repair, felt that he had hurt himself in the groin.

I perceived, on my first examination, a tumor about the size of a large marble right over the femoral artery, on the line of Poupart's ligament, which emitted to the touch a distinct, firm, unyielding pulsation. I immediately resorted to the usual methods to effect a cure, but all efforts seemed futile. The tumor having formed so high up I was unable to bring the necessary pressure above it to lessen in the least the circulation through it. It increased rapidly in size, and by its pressure upon the surrounding parts, occasioned considerable pain and numbness of the limb. December 10th, tumor was about the size of a hen's egg, and, as I said before, rapidly enlarging. I decided to operate, and on the morning of the 17th of December, the "constitutional state of the patient being good," the bowels thoroughly relieved of all fæcal matter, I had the patient placed under the influence of chloroform by Hospital Steward John Dillon, U. S. Army, and cut down to, and ligated the external Iliac artery. There was no hemorrhage; pulsation in the tumor stopped at once, closed wound with stiches and adhesive plaster, and gave ½ gr. of sulph. morphia as soon as patient had passed from chloroform. Patient did not rally very well from operation and chloroform. Circumscribed Peritonitis set in, swelling and tympany of whole abdomen. It yielded, however, readily to treatment, as follows: Hot aqueous solutions of opium to abdomen and wound; internally ¼ gr. of morphia every two hours.

December 22d, patient doing well; tympany and peritonitis subsided; passed urine through catheter up to this time; removed adhesive plaster this day and dressed wound with carbolic acid and sweet oil. Injected solution of chlor. sodæ to wound which emitted bad odor and sloughed considerably.

December 30th, patient is in fair way for recovery. Sloughing has ceased; ligature did not come away till January 9th, 1872—twenty three days from date of operation. No hemorrhage or bad symptoms followed. At this date, January 22d, wound has nearly healed; patient's general health is good; is able to sit up in bed and move about the ward on crutches.

CAMP WRIGHT, CAL., Jan. 22, 1872.

